# How effectively are social accountability mechanisms being applied in mental health services within the newly federalized health system of Nepal? A multi-stakeholder qualitative study

**DOI:** 10.1186/s12913-023-09765-1

**Published:** 2023-07-17

**Authors:** Hridaya Raj Devkota, Yuba Raj Baral, Bindu Khanal, Pratik Adhikary

**Affiliations:** 1 Institute for Social and Environmental Research Nepal (ISER-N), Bharatpur-15, Chitwan, Nepal; 2 Manamohan Memorial Institute of Health Science (MMIHS), Kathmandu, Nepal; 3 Padmakanya Campus, Tribhuvan University, Kathmandu, Nepal; 4 PHASE Nepal, Bhaktapur, Nepal

**Keywords:** Federalization, Health system, Social accountability, Mental health, Nepal

## Abstract

**Background:**

The burden of mental health problems and inequalities in healthcare has emerged as critical issues, in Nepal. Strengthened citizen-driven social accountability (SA) is an effective strategy for building equitable health systems and providing quality healthcare services to all, yet SA in mental health is an under-researched area in Nepal.

**Objective:**

This study explores changes in mental health service delivery in the re-configured federal health system and discusses the functioning and effectiveness of SA in the federalized context of Nepal.

**Method:**

This case study research used a qualitative approach to data collection. We conducted Key Informant Interviews (KIIs), and Focus Group Discussions (FGDs) with local stakeholders including people with experience of mental health problems. The audio-recorded interviews and discussions were transcribed and analyzed using a thematic content method.

**Results:**

A total of 49 participants were recruited, and 17 participated in interviews and 32 participated in six focus group discussions. From the data, eight themes emerged: Policy challenges in mental health, Governance and service delivery, Tokenism in the application of social accountability processes, Weak role of key actors in promoting accountability, Complaints and response, Discriminatory health and welfare system, Public attitudes and commitment towards mental health, and No differences experienced by the change to a federal system. It was found that existing health policies in Nepal inadequately cover mental health issues and needs. The prevailing laws and policies related to mental health were poorly implemented. There is a lack of clarity at different levels of government about the roles and responsibilities in the delivery of mental health services. Poor intra- and inter-governmental coordination, and delays in law-making processes negatively impacted on mental health service delivery. SA mechanisms such as social audits and public hearings exist within government health systems, however, application of these in mental health services was found poor. Rights-holders with mental health problems had not experienced any change in the provision of healthcare services for them even after the federalization.

**Conclusion:**

Mental health is insufficiently addressed by the health policies in Nepal, and SA mechanisms appeared to be rarely institutionalized to promote good governance and provide effective healthcare services to vulnerable populations. The provision of more equitable services and honest implementation of SA tools may foster greater accountability and thereby better service delivery for people with mental health problems.

## Introduction

Mental health problems are prevalent, posing a significant public health threat around the world [1]. However, this issue is not high on government priority lists in many low- and middle-income countries (LMICs). World Health Organization’s (WHO’s) key policy suggests to integrate mental health into primary healthcare and deliver services through a country’s existing health system [2]. The WHO Special Initiative for Mental Health 2019–2023 and Universal Health Coverage (UHC) for Mental Health 2019 [3, 4] focused on advancement in addressing global mental health issues; however, the health systems, particularly in low- and middle-income countries (LMICs) like Nepal, are reported to often fail to meet the needs of people with mental health problems [5].

In Nepal, the health system is overstretched due to the high burden of other diseases, low availability of trained health workers and financial resources, political instability, and poor governance and accountability. This mirrors the situation of other LMICs with mental health funding reported to be only a small proportion of the health budget which may be an average of 2.1% in lower-middle-income countries and 1% in low-income countries [6]. Some literature report corruption, poor governance, weak implementation of regulations, and lack of political commitment, as key factors that undermine equitable and effective healthcare services in countries like Nepal [7].

Power relationships between health system actors and accountability are central considerations to health governance [8]. Implementing social accountability (SA) mechanisms can contribute to improved governance, thereby increasing development effectiveness through empowerment and better service delivery [9]. However, enhancing the “supply side” of governance or top-down accountability mechanisms that focus on promoting the political and administrative rules and procedures, auditing, and formal law enforcement have found limited success [10]. In recent years, more attention has been given to the “demand side” of governance, or bottom-up accountability, that strengthens the voice and capacity of citizens or rights-holders and demands greater accountability and responsiveness from duty-bearers such as public officials, service providers and policy-makers [7, 11]. Rights-holders refers to those individuals and social groups who have the rights, and duty-bearers are those who have the obligation to respect, promote and realize human rights.

SA is citizen-led action to hold public officials and service providers to account for the use of public resources and services delivered. SA relies on civic engagement and ensures that duty-bearers fulfill their obligations and commitments, holding the government responsible for violating or neglecting its duty [9, 12]. It represents “the broad range of actions and mechanisms beyond voting that citizens can use to hold the state to account, as well as actions on the part of the government, civil society, media, and other societal actors that promote or facilitate these efforts” [13]. SA, as a framework for governance in the health system is gaining popularity among LMICs. Despite the increasing use of SA practices globally, there is limited evidence on its effective implementation in the health sector particularly in LMICs such as Nepal [14, 15]. For the last two decades, the Government of Nepal (GoN) has made efforts to improve service delivery at the local level, adopting legislation encompassing governance and social accountability mechanisms. The Local Self-Governance Acts of 1999 and 2007 were important breakthroughs in the devolution of power and resources to local government. They established the key foundations for local governance through the provision of participatory planning, decision-making, and services to the citizens through grassroots democracy [16, 17].

Nepal became a federal democratic republic, and in 2015, Nepal adopted a new constitution that established a three-tiered government consisting of a central federal government, seven provincial, and 753 local (municipal) governments. With the implementation of the federal structure, the country’s health delivery system changed with a new health policy. In the spirit of the new constitution, a range of laws and health acts, for example, the Public Health Act 2018, Health Insurance Act 2018, and Safe Motherhood Act 2018 were enacted. Local governments are autonomous to formulate their own laws, policies, directives, and implementation of service delivery arrangements in line with the national policy.

Federalism provides greater scope for citizens’ participation in political and policy processes and power-sharing between different levels of government, i.e. federal, provincial, and local [18]. In Nepal, it was expected to result in better governance and service delivery including reduced health service disparities, with greater accountability by duty-bearers after federalization. However, policy-making and planning processes at the provincial and municipal levels are not yet fully realized.

In the changed country structure and system, it is unclear how health policy/strategy in Nepal has addressed mental health issues, and how effectively the social accountability mechanisms are functioning to impact key government functions at different levels in relation to mental health service delivery and minimizing service gaps. What are rights-holders’ experiences (i.e. people with mental health problems) regarding social accountability in mental health services? The purpose of this study was to explore health policy/strategy and mental health service delivery mechanisms adopted by the federal, provincial and local level health facilities in two provinces in Nepal, and to discuss the functioning and effectiveness of social accountability in the health sector as it relates to mental healthcare service delivery.

## Methods

### Study design

The study applied a descriptive case-study design using qualitative approaches to data collection. We used multiple data sources including a literature review, focus group discussions (FGDs) and key informant interviews (KIIs) to enhance data credibility [19, 20]. A series of interviews and focus group discussions were conducted with a range of stakeholders that included both duty-bearers and rights-holders at different levels within the health service sector. Six FGDs and 17 KIIs were conducted from February to March 2021.

### Study context

Mental health service in Nepal was started through general hospital settings with the first psychiatric outpatient services opened in 1962 [21]. The only dedicated mental hospital established in 1984, is located in the capital city Kathmandu, in Bagmati province, and currently provides tertiary-level care with a capacity of 50 beds. Secondary, tertiary, or other specialist health facilities at provincial and national levels deliver mental health services mainly by medical colleges, provincial government hospitals, and a few private hospitals. Some non-governmental organizations (NGOs) and private sector organizations provide mental health and psychosocial care services through their clinics and community mental health programs including counseling services at local levels in collaboration with the Ministry of Health and Population (MoHP) [22, 23].

Nepal’s (first) mental health policy was implemented in 1996. Later, in 2017, the Ministry of Health and Population (MoHP) introduced the Community Mental Health Care Package − 2074 following the WHO criteria for integrating mental health into primary care and the Mental Health Gap Action Program (mhGAP) to facilitate implementation of the National Mental Health Policy and delivery of evidence-based interventions by non-specialized health workers in primary health care settings [24, 25]. The National Mental Health Strategy and Action Plan (2020) provides the basis of Nepal’s plans for mental healthcare. Based on this package, two districts of each province including Bagmati and Gandaki rolled out a community mental health program, started as a pilot intervention, with the plan for scaling up nationwide. Nepal joined the WHO “Special Initiative for Mental Health” in 2021; however, mental healthcare services are not yet fully integrated into the general health service delivery system. In terms of government financing, less than 1% (0.18% in 2018/19) of the health budget was allocated to mental health and has not increased as a national budget item [22, 26].

### Study participants and recruitment

Study participants included both duty-bearers, such as elected representatives, government officials, and service providers, and rights-holders including consumers with a lived experience of mental health problems, civil society and media representatives, and multilevel (federal, provincial and local) stakeholders. This study was a sub-study within the evaluation of a mental health mainstreaming advocacy program being undertaken in Bagmati and Gandaki provinces by a national NGO [27]. The participants were selected and recruited purposively from those two provinces, following criteria intended to include the views and concerns of all relevant stakeholders at different levels. State actors (duty-bearers) and rights-holders were recruited for interviews and discussions. Interviews were face-to-face with key informants, however, a few interviews were conducted over the phone due to COVID-19 precautions. The number of interviews and focus group discussions were determined by data saturation [28].

### Data collection tools and procedure

Interview and focus group topic guides were developed and tested before administration. The topic guides included questions related to mental healthcare service provision within the existing health system, service accessibility, and quality of care provided by the health facilities. The guide incorporated questions about social accountability tools and mechanisms adopted in the health sector and its functioning in the changed government structure. Four key elements of social accountability — citizen’s participation, transparency, monitoring, and response — were focused on in the questions. The topic guides, interviews and discussions were all in Nepali language.

Three authors, experienced in qualitative data collection, conducted the interviews and discussions with the help of a research assistant. The role of the research assistant was to obtain consent and take notes. All the interviews and discussions were audio recorded with the participant’s consent. Considerable effort was put into building rapport between participants and researcher at all stages of the data collection process to reduce any perceptions of power imbalance.

### Data analysis

After the completion of field data collection, we followed a number of steps before the interpretive phase. The first step involved transcribing verbatim all the audio recordings in Nepali and translating them into English, which was done by the second and fourth authors. The first and last authors reviewed all transcripts and interview notes, reading, re-reading, and reviewing for overall understanding; and the first author followed the open coding process and coded interview data by applying a paraphrase or label that described what was interpreted in the passage as important using RQDA software. The last author checked the coded data to ensure the meaning and consistency of the codes. Following the thematic content method, we then analyzed the data in five stages: familiarization; identifying themes; indexing; charting/mapping; and interpretation. This method allowed for the contrasting and comparing of data by themes across many cases or interviews and retained the connection to other aspects of individual accounts [29]. To ensure correctness the last author crosschecked 10% of the transcriptions, translations, and data coding to determine any sub-themes to be grouped together and any concepts identified (Table 1). Themes and sub-themes were analyzed in relation to the research questions and are described in results.

**Table 1:** Themes and subthemes by domain of analysis

Domain of analysis	Themes and subthemes generated
**Mental health policy and service delivery**	• Policy challenges in mental health o Inadequacy of current mental health policy o Poor translation of existing policy and laws into practice o Mental health is not a government priority • Governance and service delivery o Unchanged system and services in the federalized country structure o Gap between availability (resource, service) and needs
**Functioning and effectiveness of social accountability mechanism**	• Tokenism in the application of social accountability processes • Weak role of key actors in promoting accountability o Role of duty-bearers o Role of CSO, media o Role of citizens • Complaints and response
**Perception of rights-holders towards the system, attitude of duty-bearers, and experience of service use**	• Discriminatory health and welfare system • Public attitudes and commitment towards mental health • No differences experienced by the change to a federal system

## Results

We organized the result section describing the characteristics of study participants followed by the findings. The findings are presented around three domains grouping together the themes and subthemes based on their similarities (Table 1). The mental health policy and service delivery domain contained two themes: policy challenges in mental health, and governance and service delivery. The first theme consisted of three subthemes, and two in the second theme. The other two domains — functioning and effectiveness of social accountability mechanisms; perception of right-holders towards the system, attitude of duty-bearers and experience of service use — consisted of three themes for each. The findings around these themes and subthemes are summarized and substantiated by suitable interview and group discussion quotes.

### Characteristics of study participants

Out of the 49 participants, 65% were state actors (duty-bearers) and 35% were rights-holders that included rights advocates, civil society and disabled people’s representatives, and people who experienced mental health problems. About 27% of the total participants reported that they experienced mental health problems. The majority of participants (82%) were above 35 years of age, and over half (51%) were female. In terms of participants’ caste and ethnicity, more than half (53%) were Jana Jaati, 45% Brahmin and Chhetri, and 2% Dalit. In Nepal’s caste hierarchy, Brahmin and Chhetri are considered as higher caste groups, and Dalits are considered as the lowest and most oppressed caste groups. Jana Jaati is categorized as indigenous people. Only 41% of study participants had a higher level of education, about one-third had a secondary level, and 14% had no formal education (Table 2).

**Table 2:** Characteristics of study participants

Background Characteristics	Number (n = 49)	%
Participant type		
State actors (Duty-bearers)	32	65
Non-state actors (Rights-holders)	17	35
No. of participants with lived experience of mental health problems	13	27
Gender		
Male	24	49
Female	25	51
Age group		
18–25 yrs	1	2
26–35 yrs	8	16
36–45 yrs	20	41
46 yrs and above	20	41
Caste and ethnicity		
Brahmin/Chhetri	22	45
Jana Jaati	26	53
Dalit	1	2
Education		
No formal education	7	14
Primary level (< 6 grade)	6	12
Secondary level (6–12 grade)	16	33
Higher level (University)	20	41

## Policy challenges in mental health

### Inadequacy of current mental health policy

A number of study participants reported that the existing government policy did not adequately as there was no separate mental health policy was developed at a provincial or local levels. However, some of the participants reported that a few local governments have developed their own laws and policy directives based on the national policy and initiated implementation at the local level. One of the participants stated,

There is no separate policy developed at the province and local level to address mental health. But a few local authorities have allocated some budget to address the problem. At the federal level, they have developed a mental health policy that gives some guidelines in rehabilitation, treatment, and raising awareness such as using dignified words for them. Also, their voting rights, jobs and right to give candidature for election as other citizen are ensured.

- KII, Govt. official.

In the disability rights area, the lack of policy at a local level was also a barrier to addressing systemic inequity in the mental health sector. For example, a disability rights holder expressed,

To date I did not find any separate policy developed in province and local level to address mental health. We have not seen changed structure and system to address mental health issues.

- KII, OPD Leader.

Other interview participants reported that there was no standalone policy for mental health in Nepal and any existing policies that included mental health by different ministries were not comprehensive and clear enough for local-level governments to follow. It was noted that the disability care sector was governed with policies and legislation and that mental health was not included in the disability area. One of the local government authorities stated,

We have clear policy and laws to provide allowance for people with disability which is categorized into four groups according to the severity. This does not include persons with mental health problems. We allocate budget in disability and distribute allowances based on federal level policy, laws and acts.

- KII, Municipal Mayor.

### Poor translation of existing policies and laws into practice

A majority of participants spoke about the failure of various levels of government to implement and enforce any existing policies and laws relating to mental health. It was noted in a focus group discussion that the complexity of mental health sector governance and the stigma associated with it has often resulted in poor policy implementation. The participants in the health facility management committee focus group discussion uttered,

On this subject, policy or laws have not been implemented in the health action plan. In my view, health policy may be at a higher level but there is a lack of following it in practice. That health policy is not implemented here yet.

- FGD, HFOMC

In other focus group discussions, the lack of public awareness, weak advocacy, and the rights-claiming capacity of the rights-holders were also reported as reasons for the government not giving attention to the existing policy implementation. The authorities and policy-implementing agencies at the local level were felt to not be fully aware of the policy provisions for mental health. For example, a participant in a focus group discussion voiced,

It’s difficult to argue that there is no policy implementation at all but, there is a lack of public awareness about mental health in this community. It is, therefore, a public awareness campaign on this issue that should be undertaken to help people understand the problem and put the policy into practice.

- FGD, HFOMC

Other respondents noted that although there had been reforms to the rules and policies related to mental healthcare, they have not been put into practice. Some participants asserted that none of the governments at national, provincial, or municipal levels had taken any steps to mainstream and integrate mental healthcare into the primary healthcare system. Despite being expressly mentioned in the policy; it is only partially put into practice. One of the Federal Ministry’s senior officers said,

There are some changes at the policy level in the mental health service provisions over the past years at different levels. We have established the Epidemiology and Disease Control Division (EDCD) at the federal level that includes mental health. EDCD coordinates with different stakeholders in relation to mental healthcare. This is one of the good examples of progress over the last few years. We have also revised the mental health strategy developed in 1996.

- KII, Senior MoHP Official.

Some interview participants opined that mental health is a neglected issue and that the government should understand its wider implications, the need for political commitment to implement the existed policies. The informal sector rights advocates expressed,

A greater political commitment is required from local to federal level politicians. We have some solid policies to address mental health, but they are not being implemented effectively, so we have to focus on this as well.

- KII, Senior Official, INSEC.

### Mental health is not a government priority

Study participants claimed that the government had not kept mental health on its priority agenda. Respondents noted that policy and decision-makers needed to comprehend the seriousness and consequences of mental health problems at all levels and elaborated that with appropriate care, many mental health problems can be improved. A participant from the Federal Ministry of Health stated,

In the health sector, we have a good policy that covers many aspects but there are gaps in practices. The policy is not being implemented properly. We cannot see the immediate serious symptom in mental health like other acute diseases, for example diarrhea and respiratory problem. This is why it is not getting priority.

- KII, Senior MoHP Official.

Another participant expressed a similar view that the government was not working proactively to address mental health problems. He said,

Mental health problems can recover after psychosocial counseling and regular treatment. Some NGOs are working on it, but the government is not working actively to address the mental health problem.

- KII, Member of Human Right Commission.

Mental health was seen by some as a national issue that should be taken care of by the central government. One of the respondents stated,

Mental health issues are not only the problem of our municipality. This is a national problem. So, it is essential to manage budget from federal (central) level and vital to formulate national mental health policy to minimize resource and service gaps in mental health. If it is not possible to manage from central level only, resource sharing could also be possible.

- KII, Municipal Mayor.

## Governance and service delivery

### Unchanged system and services in the federalized country structure

Participants reported that they saw the structural changes and power delegation to different levels of governments in the federal system; however, the changes have not materialized as expected, particularly in the system development and service provision to people with mental health problems. One of the government authorities at the provincial level stated,

In our three tiers of the government system, there are district hospitals, Primary Health Centers, and health posts at a local level with its staff having basic knowledge of mental healthcare. They do not have trained mental health specialists. The government has made some efforts to provide mental healthcare through the primary care system, but it has not materialized yet.

- KII, Senior DoHS Official.

Although the health policy has provisioned the mental health desk at provincial and district level hospitals and separate mental health units at all health centers, it was not yet established. One of the local-level government authorities said,

I don’t see any mental health desk (unit) in district or even in provincial level hospitals. There are one or two centers that offer mental health services in Pokhara and a few hospitals in Kathmandu that offer mental health services. Indeed, it is essential to establish a separate mental health unit in each health center countrywide. Federal government should take responsibility for this.

- KII, Municipal Mayor.

Unclear roles and responsibilities across governments and inadequate intra- and inter-governmental coordination at various levels, have resulted in delays to policy implementation and effective service delivery. For example,

Local-level governments are responsible for planning, coordination and reporting to higher level, but this practice is very poor in the present situation. In many cases, there is no clear definition of who is responsible for a certain task. The role of local, province and federal government is still unclear.

- KII, Senior MoSD Official.

### Gaps between availability (resource, service) and needs

Respondents reported that there was a slight increase in the amount of money spent on mental health from the general health budget in the study year compared to the past year. Similarly, it was noted a small number of local governments allocated some resources within the broader health sector to mental health. In addition to financial resource constraints and gaps, respondents raised issues of limited trained mental health workers to provide mental health services. One of the respondents at a tertiary-level hospital said,

Since the last few years there are several changes like an increase in budget from the federal level, and mental health is now kept within the primary healthcare package, but the changes are not sufficient. I do not have a lot of experience in policy-making so I do not have much insight, but I can see little change in this area.

- KII, Health Provider.

Another respondent added,

The process of including psychotropic drugs in the essential medicine list is moving forward as a policy with the intention of providing medicines free of cost to patients with mental health problems. But I have not seen any concrete plan and policy of the government to narrow down the gap between required and available trained human resources in mental health.”

- KII, District Public Health Officer.

Participants identified that a wide gap existed not only between the availability of resources and needs but also in the treatment, rehabilitation, and promotion of mental health. For example, one interviewee expressed,

Anecdotal evidence suggests that 20–25% of the population are suffering from at least one type of mental illness. Out of total patients, even less than half are not getting medicine in mental health.

He further added,

“We don’t have enough psychiatrists in the country, and there are no psychiatrists in district hospitals. Health staffs have just basic knowledge of mental health. Only a few selected staffs had mental health training. As a result, the patients have not been able to complete the treatment on time. Another problem is the unavailability of medicine to buy at the local level.

- KII, District Public Health Officer.

Similarly, another respondent said,

We have very few mental health hospitals in Nepal, and their services exist in the big cities. It is important to expand mental health services all over the country. It is better to establish a mental health hospital in each municipality. If not possible at least at district level to minimize the current service gaps.

- KII, Municipal Mayor.

Furthermore, participants reported that mental health preventive, promotive, and rehabilitative services in mental healthcare are nearly absent at the community level despite the high need for them. Integrating mental health services into primary healthcare has not been effectively done. It was also stated that recently the Nepal government had started a program for rehabilitation and support of a range of target groups that require social support and protection including people who are homeless and with mental health disorders. One of the participants stated,

I found mental health issues and their rehabilitation have created complexity at the local level. There are a few rehabilitation centers and also a “Manav Sewa Ashram”. We rescue the helpless including people with mental health problems and bring them to the “Ashram” (rehabilitation home). They do not have any regular source of funds and are struggling to offer services and manage the centers. We supported them with some budget from local level which is insufficient.

- KII, Municipal Mayor.

## Tokenism in the application of social accountability processes

A majority of participants reported that there was some sort of accountability mechanism developed and that a number of tools had been introduced in the government system. Participants named these as including the citizen charter, public information board, complaint box, social audit, public hearing, and participatory planning. However, the application of those instruments was reported to be very poor. Participants also reported that mental health issues were rarely included while applying these tools. One of the government officials said,

It is important to bring local people and authority in the planning process to the policy-making. Local authorities have good knowledge about the problem, and the issues must come from the local level. In the planning process, we have to follow the bottom-up approach. Some efforts are made to follow this, but it is not working properly due to several reasons.

- KII, Senior MoSD Official.

It was also reported that people with mental health problems were systematically excluded from participating in the planning, monitoring, and decision-making processes. One of the respondents stated,

I don’t think people with mental health problems participate in the planning and decision-making process. I have found that blind persons and people with physical disabilities participate in NGO/INGO programs. But they are participating in the government program. Occasionally, I can see people with physical disability in some events, but persons with mental health problems are not participating.

- KII, District Public Health Officer.

Some of the respondents expressed that the participation of persons with experience of mental health problems was never ensured in the provincial level planning process. It included different levels of stakeholders and experts, but the representation of vulnerable groups such as people with a lived experience of mental health problems was often ignored. For example,

Provincial level planning involves different level stakeholders. But these particular issues are basically focused on policy at the federal level of the health system. In the current system, there is no local-level involvement in provincial program and policy process.

- KII, Senior Govt. Official.

A majority of interview participants reported that there was no transparency and consistent information flow at all levels of government. It was stated that the government had a designated information officer in every public office; however, the information they provided was often inconsistent and unreliable. Advocates and journalists consistently reported difficulty to get factual information from government offices. On the other hand, a few government representatives argued that they were transparent in program and budget noting that program and budget information were posted on the official website, and the budget expenditure would be overseen by a committee that includes beneficiary representatives. One of the respondents described,

We inform the public about the available budget for the program and approved projects through our red book. The plan and budget are kept on the website as well, and anybody can access it. In the case of a construction project, we display the information on-site. After completion of the project, HFOMC (in the case of the health budget and another committee such as the user’s group) evaluate the program and budget expenditure. Moreover, we organize social audits and include the feedback in the next project implementation and expenditure.

- KII, Municipal Mayor.

## Weak role of key actors in promoting accountability

### Role of duty-bearers

Participants stated that they expected better governance, more efficient service delivery, and greater accountability as a result of the changes in the government structure with power delegation. They report there was an uptick in criticism of public service providers with the negative attitude of duty-bearers remaining unchanged. One of the participants expressed,

There is no fundamental change observed in the attitude of government officials and noteworthy progress on governance and accountability in the changed scenario as well. There is a problem in the practices and implementation of activities as specified in their roles. For example, it is mandatory to have a social audit or public hearing by all public service offices, but they do not follow it. Many of them are implemented as a formality only to meet the requirement.

- KII, Journalist.

Another participant added,

Out of the total, only 10–15 local municipalities may have been attempting to maintain good governance and a transparent system. In the present context, there is high corruption and misuse of resources at the local level. Provincial government seems ineffective in its role and responsibility.

- KII, Rights Advocate.

Participants raised concerns around intra-departmental and external monitoring and supervision systems that were not functioning properly and effectively noting there were no clear guidelines for performance monitoring in the current changed structure. The integrity of authorities was also raised repeatedly by the participants. The local representative stated,

There is a lack of monitoring system from both government and public level. The low involvement of people in the development process is also a concern. They feel this is the government’s job. We also do not seek the reasons why the work is not performed in time as per plan. In most cases, there is a lack of accountability at both public and government levels.

- KII, Ward Chair.

### Role of civil society organizations and media

The role of civil society organizations (CSOs) and media is very important in keeping duty-bearers accountable. But the enactment of this role was reported as weak and ineffective in the study area. Some claimed that their role focused on their own interest and in support of power holders rather than working in favor of the general public and voiceless. One of the participants claimed,

Media can bring changes in the community through the flow of information. It can help to build up trust in the community toward developmental work. But like in other sectors, there is a lack of professionalism in the media as well, and it is not able to play an effective role in this issue. Moreover, investigative journalism is challenging and lacks willingness. It is easier for us to sell the news than investigative information.

- KII, Journalist.

Another participant had a similar view,

“Monitoring system is poor in our context. There are systems in the country, but they are not followed properly, resulting in increased corruption and human rights violation day by day. We have many rights organizations and associations such as bar associations, federations of journalists, NGOs/CSOs, and human rights organizations, but still we are not able to work effectively to make duty-bearers accountable for the promotion of rights of the vulnerable populations such as persons with mental health problems.”

- KII, Member of Human Right Commission.

Participants working with government authorities explained the importance of the role of citizens and community actors for the effective application of social accountability mechanisms. Citizen’s participation, and the active and constructive role of civil society organizations including constant monitoring and managing complaints could improve accountability and building trust between duty-bearers and rights-holders. One of the participants explained,

We organize meetings with the community people and inform them about the program process, achievements, and expenditure. We also publish in newspapers and broadcast from FM radio about our activities and put the notice in public places seeking public participation, particularly in the planning process, but very few people take interest. We have a complaint box in our office, but people rarely use it. Nonetheless, we have continued information flow and also conduct public hearings including different stakeholders.

- KII, Ward Chair.

### Role of citizens

Participants, including the representatives of people with disabilities, described being prevented from participating in local planning and decision-making processes. The representation of different groups, such as the National Federation of Disabled, are invited to participate in local processes, but the existence and representation of people with psychosocial disabilities are neither recognized by the government system, nor by the federation itself. A representative of disability organization claimed,

While talking about our participation in the local planning and decision-making process, it is an increasing trend to invite us at different stages. However, the representation of people with mental health or psychosocial disabilities is not formalized yet.

- KII, OPD Leader.

Citizens may be unaware of the public procedures, services, and entitlements that they have the right to receive from the government. According to respondents, the administration has created no space or area for citizens to participate in policy and decision-making, rather the government system discourages the participation of excluded groups. In line with other participants’ claims, one of the government officials said,

We have to accept that all government offices and their system are not very transparent, and the general public is not informed about the public issues. Many people do not keep concerned because they don’t know the issues. Also, there is no system, and it is difficult for them to get the information if somebody takes an interest. Transparency and information giving are serious issues in our practice.

- KII, Senior Govt. Official.

## Complaints and response

Respondents noted a system of “complaints and response” with various tools was established in public offices. A complaint box, complaint register, toll-free telephone, and a designated officer for taking and responding to complaints were reported the most common tools provisioned. However, a majority of participants stated that they were not functioning well. One of the participants expressed,

Reporting and complaint-taking procedures are there, but they are not effective. Very few people go there for reporting. This may be because many people don’t know there is a reporting/complaint system or they don’t trust that they will get a response.

- KII, OPD Leader.

One participants from a provincial ministry acknowledged the fact that the response system was poor and discriminatory. He said,

Our response system is very poor. I think people lack trust in the government system, and the people, particularly the excluded groups, do not get services in time and in an effective way. People in general have not complained that they are not getting a response from the authorities. But some people with mental health problems reported to me that they often get negative responses. So, there is discrimination in response to people with a mental health problem.

- KII, Senior Govt. Official.

Another participant stated,

We do not have a complaint box or any system of taking complaints in our health facility. I have seen a complaint box installed in ward offices, but not in the health facilities. It may be because some issues cannot be addressed directly. If it functioned properly, it may help to improve services.

- FGD, HFOMC

## Discriminatory health and welfare system

Study participants, with a lived experience of mental health problems, reported being systematically discriminated against, and that some laws and policies prevent them from receiving services. Respondents reported that the government provided cash allowances to people with disabilities, but those with psychosocial disabilities (people with a mental health problem) were excluded from this. It was also reported that mental healthcare services were not included fully in the primary care healthcare system. One respondent stated,

There is a need to change the existing discriminatory policy and laws against people with mental health problems. Discriminatory laws related to property rights, employment, right to health or other fundamental rights of citizens provided by the constitution are still prevailing.

- KII, Human Rights Advocate.

## Public attitudes and commitment towards mental health

Participants reported that people with mental health problems often face disrespect and humiliation in society and while receiving services. They perceived negative attitudes and behaviors of service providers. One participant expressed,

People look at us differently, not only in the community but also while seeking services. There is stigma, negative beliefs towards us, and people call us using undignified words.

- FGD, Self-help Group.

Participants further stated that healthcare providers and officials in government offices avoid them, not listen to them and often ignored. Some reported that they often faced rude and impolite behaviors, including from higher level government officials. A government official admitted,

We have realized the issues in this sector and try to address them from our level. The undignified words previously used in policies and other official documents are now changed. However, it may take a long time to change the individual attitude and behaviors.

- KII, Senior Govt. Official.

Prevailing negative attitudes towards mental health among the general public, including policy-makers and service providers, are some of the key barriers reported by participants for the effective application of accountability tools for the promotion of the rights of people with mental health problems. They are not considered a citizen and are prevented from accessing their basic rights. The participant argued,

People have a misunderstanding. They do not consider mental illness to be a curable disease. It is understood that the disease is caused by witchcraft, ghosts, etc. People prefer to consult spiritual and traditional healers rather than medical consultants. There is a social stigma about mental health, and they often hesitate to consult mental health experts even if they have the problem.

- KII, District Public Health Officer.

Participants noted that there was no culture of proactive initiation and support to service users from the government authorities. One high-ranking official at the federal level ministry expressed,

Even the duty-bearers do not inform people of their duty. I do not claim that there is a citizen charter at every office. We have to inform people what kind of services are there, and we need to communicate clearly, which helps to build trust between service providers and service users. But it is not happening. We have trained and qualified human resources, but we need to change our attitude and behavior to improve this.

- KII, Senior MoHP Official.

## No differences experienced from the change to a federal system

Participants reported that they did not experience any differences in relation to policy, systems, and service provision since the roll out of the National Mental Health Strategy and Action Plan 2020. It was the opinion of some respondents that their participation was not ensured in the policy development process even with the new system. Participants discussed issues related to their basic rights and health services were not covered in the changed policies and health system. A disability rights advocate expressed,

There are no fundamental changes in the response system overall. Attempts are made to ensure voting rights to meet the political interest of politicians. Other than that, there are no systematic changes to respond to people’s issues.

- KII, Disability Rights Activist.

Respondents associated with government authorities and advocacy services noted there were some indications of change in policy, services, and attitudes of government authorities; however, change was slow and not as expected by the people. One of the respondents said,

There are some changes in public health service acts and rehabilitation acts to provide equal access to healthcare for people with mental health problems. Another example is the use of dignified words for people with mental health problems, but these changes are not sufficient. Moreover, the national mental health act is endorsed, and we were also involved in that process.

- KII, Senior Govt. Official.

Focus group participants reported that they had not experienced any difference in health services but they had observed some structural and system changes and decentralized power due to federalization. However, respondents reported their experience relating to access to mental healthcare, service provider accountability, and attitudes towards people with psychosocial disabilities, were unchanged.

We all know that the service provision in mental healthcare is not improved with the changed structure. In our area, mental health patients are receiving their basic medicines through a program run by an NGO. It is not available in government health facilities. Some people go to Pokhara or Kathmandu to get their medicines. We had the expectation of receiving services from local health facilities after federalization, but this is not the case.

- FGD - Self-help Group.

## Discussion

This study aimed to explore changes in mental health service delivery in the federal health system and discuss the functioning and effectiveness of social accountability at different levels and learn about the experience of rights-holders. The findings suggest that the existing National Health Policy of Nepal 2019 does not adequately cover mental health issues. A separate standalone National Mental Health Policy 2017 drafted by the Federal Ministry of Health to replace the first Mental Health Policy 1996 was intended to scale up mental health activities making mental health a priority health agenda. However, it is not yet tabled for Cabinet approval. As an alternative, the Mental Health Strategy and Action Plan 2020 was recently endorsed by the government in Nepal [30]. In the federal system of Nepal, all three levels of government have the constitutional power to develop policies, enact laws, prepare budgets, and mobilize their own resources according to their needs and priorities. However, none of the provincial and local governments have initiated the process to formulate mental health policies and plans. Even with the realization by both state and non-state actors that mental health is a serious public health issue, it is still not a priority agenda of government at any level. This has resulted in delayed policy formulation and poor application of existing mental health strategies and plans. The government authorities at the policy level were not proactive in addressing mental health issues. It could be explained that the policy and system ambiguity, the inadequately defined roles and responsibilities of different levels of government for the delivery of health services, and also the complexity of the health system and mental health problems may have contributed to this [31]. Existing laws, policies, and practices were found not to be fully in line with human rights principles of non-discrimination, participation, and equity of access to services. Studies in South Asian countries report similar findings of policy gaps and poor implementation of existing policies [32].

Duty-bearers at all levels referred to a lack of specialized provision and workforce for mental health. Respondents cited a lack of available options for high-level intervention and medicine as barriers to responding to the mental health needs of the Nepali population. However, respondents also noted many common mental health conditions and needs can be managed and supported effectively by non-specialized and primary healthcare services. This gap between resource and service availability, and the needs of health consumers is also reported by researchers in other low-income countries [5, 33, 34].

The constitution of Nepal has given a larger role in service delivery, semi-judicial, and fiscal authority to local governments. It has also created greater responsibilities for equitable, fair and effective service delivery through shared power and resources, improved governance and accountability toward citizens. However, this study did not find any indication of improved governance and accountability in mental health service delivery in the restructured and federalized health system. The past studies in different contexts reported mixed results of decentralizing power to provincial and local governments with a restructured health system. The study conducted in a high-income country (Canada) showed that shifting executive power to provincial and local levels has positive effects on local resource usage through participatory bottom-up planning, increased accountability and reduced bureaucracy in decision-making [35]. In contrast, some other studies conducted in LMICs such as Ghana, Zambia, Uganda, the Philippines, and Pakistan suggested that similar reforms exacerbated inequalities, weakened local commitment to priority health issues, and interfered with service delivery in some points [36–38]. This suggests that the prospective gains with a restructure and power shift to provincial and local levels are contextual, and not guaranteed.

Despite the efforts being made by both the government and civil society in Nepal to promote SA in the health sector through the use of “mechanisms” (structures, tools, and activities / processes), such as health management committees, social audits and community health scoreboards (CHSBs), the study sketched a grim picture of the accountability landscape with the ineffective functioning of those mechanisms. It was also revealed that while mental health policy making and planning take place mainly at the federal and provincial levels, participation and social accountability mechanisms only guarantee engagement with rights-holders at the local level. This current study found that consumers of mental health services were regarded as passive recipients of the services leading to limited citizen engagement, a weakened role of media and civil society organizations to influence mental health policy formulation and implementation. On the demand side of social accountability, we found that even though health service users were aware of their rights and expectations in healthcare, they lacked effective channels through which to voice their concerns and complaints and hold duty-bearers accountable. This finding is consistently reported by other studies conducted in Malawi, and West and Central Africa [39, 40].

Rooted in the Constitution of Nepal, some efforts have been made to promote political commitment to more accountable and transparent governance. However, the attitudes of citizens and duty-bearers towards each other reflected the negative with entrenched power structures, creating an environment of distrust [41]. This resulted in perpetuation of the previously weak roles of both citizens and duty-bearers in the application of social accountability mechanisms to promote and improve mental health services.

Another important finding of this study is that people with mental health conditions often perceived systematically discrimination by different levels of government, not only in terms of access to appropriate healthcare, but also in relation to entitlements and social security allowances provided by the state to people designated as vulnerable. Negative attitudes of service providers and society towards mental health continue to create barriers to people accessing a range of services. Previous studies conducted in Nepal support the results from focus group discussions in this study that healthcare provider’s negative attitudes often create barriers to vulnerable groups in receiving healthcare services [42], and that even if received, the quality of services provided was poor [43].

### Policy and practical implications

This study contributes to an understanding of the accountability and responsiveness of rights-holders and duty-bearers in mental healthcare services in the federal health system of Nepal and provides insight into barriers and opportunities for strengthening social accountability. The findings affirm that accountability, governance, and mental health services have not shown indications of positive change since the start of the federal system. It provides an alert that a wider discussion is urgently needed about the effective implementation of social accountability in the health sector particularly in regard to mental health. In order to provide more equitable and acceptable healthcare services to all, policy and decision-makers at all levels of government need to develop more coherent, authoritative, and responsive accountability processes.

### Study limitation

This study attempted to cover a broad area that comprised policy issues in mental healthcare services, accountability, and rights-holders’ experience of receiving mental health services in the federalization process. All three elements are important to assess comprehensively and in-depth and to understand issues related to mental health service delivery, quality, and mechanisms of redress. Nonetheless, one study is not enough to adequately explore these topics in-depth. Also, despite the steps taken to ensure quality in data collection, there could be some desirability bias as the data collection was combined with evaluation interviews of a mental health advocacy project. Developing an understanding of causal linkages between different factors and outcomes was not possible due to the qualitative nature of the study, and also the findings may not necessarily represent the whole country as this study was conducted in the selected districts of two provinces only.

## Conclusion

This study provides an overview of the current scenario of mental health policy implementation and stakeholders’ accountability and responsiveness to mental health services in the federal context of Nepal. It identifies disparities and gaps in the translation of policy into practice, the legal framework, and accountability mechanisms within the mental health sector. Mental health is inadequately covered in health policies in Nepal, and social accountability mechanisms appeared to be rarely institutionalized to promote good governance and provide effective healthcare services to vulnerable populations. Building trust between rights-holders and duty-bearers is urgently needed to promote social accountability thereby improving mental healthcare services in Nepal. Further advocacy is required to ensure that mental health receives priority on the government agenda and in future country health strategies to accelerate progress towards achieving the Sustainable Development Goals (SDGs).

## Data Availability

The datasets used and/or analyzed during the current study are available from the corresponding author on reasonable request.

## Abbreviations

DoHS: Department of Health Service
GoN: Government of Nepal
HFOMC: Health Facility Operation Management Committee
LMIC: Low-and Middle-Income Country
MoHP: Ministry of Health and Population
MoSD: Ministry of Social Development
OPD: Organization of People with Disabilities
SA: Social Accountability
